# BCMA-targeted bortezomib nanotherapy improves therapeutic efficacy, overcomes resistance, and modulates the immune microenvironment in multiple myeloma

**DOI:** 10.1038/s41408-023-00955-y

**Published:** 2023-12-11

**Authors:** Debasmita Dutta, Jiye Liu, Kenneth Wen, Keiji Kurata, Mariateresa Fulciniti, Annamaria Gulla, Teru Hideshima, Kenneth C. Anderson

**Affiliations:** Department of Medical Oncology, Dana-Farber Cancer Institute, Harvard Medical School, Boston, MA USA

**Keywords:** Targeted therapies, Drug development

## Abstract

Bortezomib (BTZ) is a standard-of-care treatment in multiple myeloma (MM); however, adverse side effects and development of resistance limit its long term benefit. To improve target specificity, therapeutic efficacy, and overcome resistance, we designed nanoparticles that encapsulate BTZ and are surface-functionalized with BCMA antibodies (BCMA-BTZ-NPs). We confirmed efficient cellular internalization of the BCMA-BTZ-NPs only in BCMA-expressing MM cells, but not in BCMA-knockout (KO) cells. In addition, BCMA-BTZ-NPs showed target-specific cytotoxicity against MM cell lines and primary tumor cells from MM patients. The BCMA-BTZ-NPs entered the cell through receptor-mediated uptake, which escapes a mechanism of BTZ resistance based on upregulating P-glycoprotein. Furthermore, BCMA-BTZ-NPs induced cell death more efficiently than non-targeted nanoparticles or free BTZ, triggering potent mitochondrial depolarization followed by apoptosis. In BTZ-resistant cells, BCMA-BTZ-NPs inhibited proteasome activity more effectively than free BTZ or non-targeted nanoparticles. Additionally, BCMA-BTZ-NPs enhanced immunogenic cell death and activated the autophagic pathway more than free BTZ. Finally, we found that BCMA-BTZ-NPs selectively accumulated at the tumor site in a murine xenograft model, enhanced tumor reduction, and prolonged host survival. These results suggest BCMA-BTZ-NPs provide a promising therapeutic strategy for enhancing the efficacy of BTZ and establish a framework for their evaluation in a clinical setting.

## Introduction

Multiple myeloma (MM) is an incurable cancer of the plasma cells that is usually accompanied by severe immune deficiency and dysfunction [1, 2]. Bortezomib (BTZ), one of the most commonly used therapeutic agents for MM, is a proteasome inhibitor that selectively inhibits the 26S proteasome, a large protein complex responsible for degrading and recycling misfolded or damaged proteins. Proteasome inhibition leads to a toxic accumulation of proteins that activates apoptotic pathways, cell cycle arrest, and ultimately, cell death [3, 4]. Though MM cells are particularly sensitive to proteasome inhibition, many healthy cells also suffer. BTZ causes severe systemic toxicity in patients, such as diarrhea, vomiting, nausea, and peripheral neuropathy, which has caused the NCI to recommend a reduced dose [5, 6]. In addition, despite BTZ’s efficacy, resistance and relapse appear inevitable. The P-glycoprotein (PgP) pump, a transmembrane protein responsible for immediate drug efflux from cancer cells, is one mechanism of BTZ resistance [7–10].

We hypothesized that using nanoparticles to deliver BTZ would slow down drug efflux, since the nanoparticles would use receptor-mediated endocytosis to deliver the drug to the inner cytosol, far away from the membrane-located PgP pump. Furthermore, a substantial portion of BTZ’s efficacy comes from its triggering immunogenic cell death (ICD), which is a mode of cell death in which the dying cell releases damage-associated molecular patterns (DAMPs) that stimulate anticancer immunity [11–13]. Recent studies have demonstrated that nano-designed drug delivery systems modulate the tumor immune microenvironment to achieve advantageous outcomes [14–16]. Using nanoparticles to deliver BTZ could effectively upregulate ICD induction by providing sustained, low-dose drug release [17–19].

To target nanoparticles carrying BTZ to cancer cells, we would need to decorate the nanoparticle with ligands that attach to the MM cell. B cell maturation antigen (BCMA) is specifically expressed on the plasma cell surface during all stages of disease progression [20–22]. Here, we packaged BTZ with FDA-approved biodegradable and biocompatible polymer PEG-PLGA nanoparticles and conjugated anti-BCMA antibody to the nanoparticles’ surface. BTZ has been successfully loaded into nanoparticulated drug delivery systems [23] and also used as a targeted therapy [24]. However, here for the first time, we investigated the impact of nanoencapsulated BTZ on the MM immune microenvironment, on drug-resistant cells, and on in vivo tumor tissue in MM NSG mice using live-localization. We hypothesized that BCMA-targeted BTZ nanotherapy would target the tumor tissue, amplify the anti-tumor effect and enhance the immune therapeutic efficacy of the free drug in the MM tumor microenvironment, as well as overcome PgP-mediated drug resistance, with minimal toxicity to normal cells.

## Materials and Methods

### Multiple myeloma cells from patient samples and normal donors

Bone marrow mononuclear cells (BMMCs) isolated from MM patient samples and normal donor peripheral blood mononuclear cells (PBMC) were procured after written informed consent in accordance with the Declaration of Helsinki and under the approval by the Institutional Review Board of the Dana Farber Cancer Institute. BMMCs and PBMCs both were first isolated by Ficoll-Paque PLUS (GE Healthcare), followed by PBS washing. CD138 + MM cells were separated from BMMCs using magnetic microbeads (Miltenyi Biotec) with CD138 positive selection [25]. CD138-negative BMMCs were cultured in complete RPMI 1640 medium supplemented with 1× antibiotic-antimycotic for 4 to 6 weeks to generate long-term BM stromal cells (BMSCs).

### BCMA-conjugated BTZ nanoparticles synthesis

Bortezomib was encapsulated with the FDA-approved PEGylated PLGA polymer, Carboxylic acid-poly(ethylene glycol)-b-poly(lactide-co-glycolide), at a ratio of PLGA (50:50);MW:20K-PEG(MW:5000) with a modified double emulsion-solvent technique, as previously described [26, 27]. Detailed method and characterization of the particles described in supplementary file.

### Development of Mo-DCs and T-cell proliferation assay

Monocyte-derived dendritic cells (Mo-DCs) were produced from the peripheral blood monocytes of healthy donors by positive selection using CD14+ magnetic beads (Miltenyi Biotec). The isolated monocytes were cultured for 6 days with Mo-DC differentiation medium containing GMCSF and IL4 (130-094-812; Miltenyi Biotec) to develop dendritic cells. Naive T cells were isolated using the Pan T Cell Isolation Kit (Miltenyi Biotec) from negatively selected CD14− PBMCs from the same donors and stored at -80°C. Once DCs matured, T cells were then revived, stained with CellTrace CFSE Cell Proliferation Kit (Thermo Fisher Scientific), and then co-cultured with untreated, free drug, or different nanoformulated pulsed MM cells for 5 days. Cells were stained with 7AAD, CD3-BV711, and CD8-PECy7. T cells were first gated for CD3-positive and CD8-positive cells. Finally, proliferating T cells were gated as CFSE_low_ population.

### Flow cytometry and confocal microscopy-based assay

All surface staining of experimental cells was performed as previously described [28]. The cells were analyzed by Fortessa-II flow cytometer (BD Biosciences, San Jose, CA). Data analysis was performed using the Diva software (BD Biosciences). Detailed methods are described in the supplementary file.

### Xenograft model for evaluation of therapeutic efficacy and off-target toxicity measured by HE staining

For the MM plasmacytoma mice model, we subcutaneously injected 5 × 10^6^ MM. 1S cells dissolved in 1:1 PBS and matrigel mixture into 6-week-old NOD Cg-Prkdcscid Il2rgtm1Wjl 9 /SzJ (NSG) female mice (Jackson Laboratory, Bar 10 Harbor, ME, USA). Once the tumor volume reached 100 to 150 mm^3^, 5-6 xenografted mice were randomly divided into each of the four treatment groups: (i) Vehicle Control, (ii) free drug BTZ (1 mg/kg once a week), (iii) non-targeted BTZ-loaded nanoparticles with equivalent amounts to 1 mg/kg (once a week), and (iv) BCMA-BTZ-NPs with equivalent amounts to 1 mg/kg (once a week). Detailed methods are described in the supplementary file. Technical staff members involved in determining euthanasia time point were blinded to the experimental purpose of the study. At the end of the experiment, major organs, including liver, lung and kidney, were isolated from all treatments along with the cancer control group that received no treatment and also from normal mice. After the preparation of tissue histology slides, HE staining was performed to compare the effect of different treatment groups by analyzing pathological changes in tissue morphology [29]. All animal studies were executed under a protocol approved by the DFCI Institutional Animal Care and Use Committee (IACUC) guidelines. Kaplan-Meier method was used to estimate mouse survival.

### In vivo real-time whole body and ex vivo organ fluorescent imaging

For fluorescent imaging, 5 or 6 randomly selected MM plasmacytoma mice per group were injected intravenously with non-targeted and BCMA-BTZ-NPs loaded with DiR NIR dye. After 12 h, 24 h, and 48 h, the mice were imaged to detect fluorescence signal homing in the whole body using the In-Vivo MS FX PRO fluorescent imaging system (Bruker, Billerica, MA) at Ex/em of 748/780 nm. Animal facility technical staff members involved in imaging were blinded to the experiment design. Following 12 h, 24 h, and 48 h, tissues were extracted from each mouse and imaged in the In-Vivo MS FX PRO fluorescent Bruker imaging system.

### Statistical analysis

All experiments or measurements were independently performed at least three times with biological triplicates unless otherwise specified. The data are represented as mean ± standard deviation with statistical significance levels: not significant (ns), *p < 0.05, **p < 0.01, ***p < 0.001, ****p < 0.0001. The results were analyzed using a Student’s t-test (two-tailed, unpaired) or one-way ANOVA followed by Tukey’s post hoc test using Graph Pad Prism software (Graph Pad Prism Software Inc, San Diego, CA, USA). Images, bar diagrams, and graphical representations were developed using Graph Pad Prism software, Adobe Illustrator, GIMP 2.10.30, ImageJ, Flow Jo, Origin 2021, and Python (version 3.10.6) with Matplotlib package (version: 3.7.1). All the graphics were created using Biorender.com.

## Results

### Characterization of BCMA-BTZ-NPs and evaluation of targeting specificity towards MM cells by cellular uptake

**Fig. 1:**
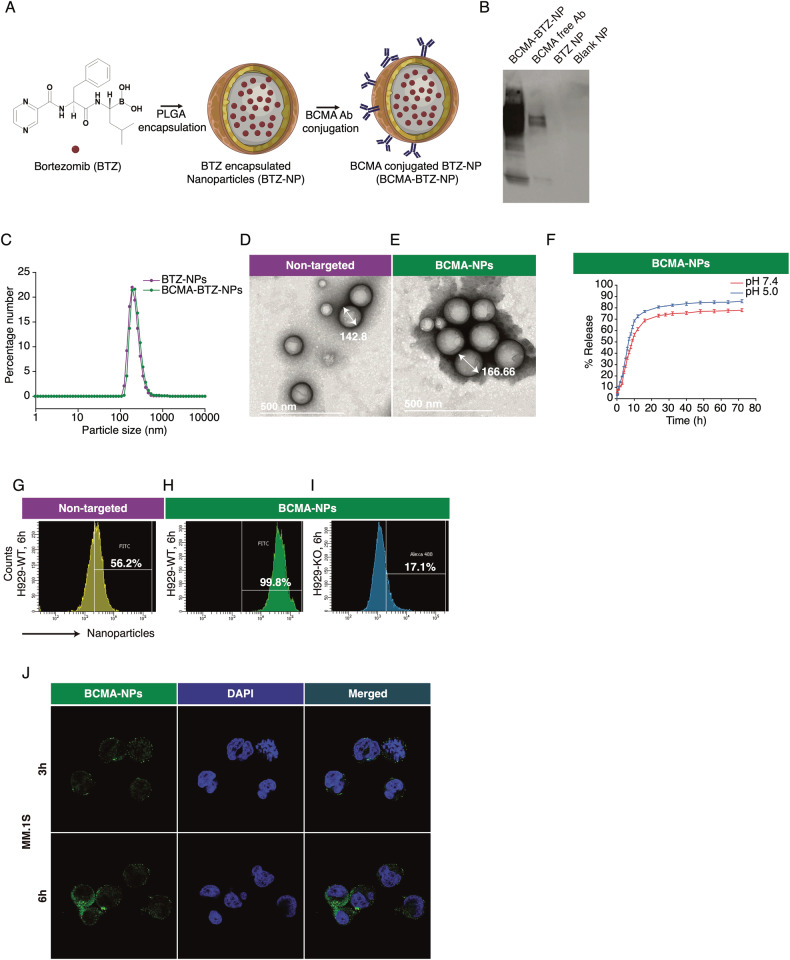
Characterization of BCMA-targeted bortezomib nanoparticles and determination of targeting specificity by cellular uptake. **A** Schematic representation of BTZ-loaded BCMA-conjugated nanoparticles. **B** Western blot image showing successful conjugation of the antibody with nanoparticles forming a smear in the 1^st^ lane, free antibodies showing bands at respective MW, as well as unconjugated and blank nanoparticles without bands in the 3^rd^ and 4^th^ lane, respectively. **C** Particle size distribution of unconjugated and BCMA-BTZ-NPs measured by DLS. TEM images represent surface morphology for **D** Non-targeted nanoparticles and **E** BCMA-BTZ-NPs. **F** In vitro drug release kinetics of BTZ-loaded nanoparticles at pH 7.4 and acidic pH 5.0. Percentage cellular uptake monitored in WT-H929 cells after 6 h of incubation with **G** non-targeted nanoparticles and **H** BCMA- targeted nanoparticles. **I** Percentage cellular uptake monitored in BCMA-KO-H929 cells after 6 h of incubation with BCMA-BTZ-NPs. **J** Cellular localization of BCMA-BTZ-NPs observed in MM.1S cells by confocal microscopy at 3 h and 6 h. Data represents one of 3 replicates of independently performed experiments. Mean ± SD, *n* = 2-5 independent experiments in triplicate technical repeats.

We first developed BTZ-encapsulated NPs using the FDA-approved biodegradable polymer PLGA, and then conjugated anti-BCMA antibodies to the COOH groups present on the nanoparticle surface using a coupling reaction (Schematic representation, Fig. 1A). We confirmed successful conjugation using protein gel electrophoresis and mass spectrometry (Fig. 1B and Supplementary Fig. 1A). Additionally, a protein assay found approximately 20 µg of surface-bound antibody for 1 mg of nanoparticles. Using dynamic light scattering (DLS), we observed that the size distribution of the antibody-conjugated particle (named BCMA-BTZ-NP) resembled that of non-targeted nanoparticles, having an average size of 154 ± 13 nm for BTZ-NPs and 168 ± 15 nm for BCMA-BTZ-NPs (Fig. 1C) with surface charges of −8.0 ± 2.5 mV and −13 ± 2.0 mV, respectively. Corresponding transmission electron microscope (TEM) images confirmed the particle size and the smooth, spherical shape (Fig. 1D, E), suggesting the particles would be compatible as a drug delivery vehicle for efficient cellular internalization. They had high drug-loading efficiency (8.3 ± 0.5% and 8.0 ± 1% for BTZ-NPs and BCMA-BTZ-NPs, respectively), and BCMA-BTZ-NPs had an entrapment efficiency of 88.6%. In terms of drug release, we found a slow and sustained release profile in PBS (pH 7.4, similar to blood), with a plateau at 24 h. At pH 5, which mimics the acidic tumor microenvironment, we observed a spike in drug release, suggesting a faster release of BTZ (Fig. 1F). Additionally, these nanoparticles were stable over 60 days in colloidal suspension under refrigeration (Supplementary Fig. 1B, C).

We next investigated the targeting specificity of BCMA-BTZ-NPs towards MM cells by monitoring intracellular uptake using flow cytometry. BCMA-BTZ-NPs had significantly more cellular internalization (99.2 ± 0.7%) than non-targeted particles (57.7 ± 0.8%) in wild-type (WT) H929 cells (*p* < 0.0001) after 6 h of incubation, with a time-dependent increase. Importantly, cellular internalization of the BCMA-BTZ-NPs in BCMA-KO H929 cells (15.9 ± 2.5%) was significantly depleted (*p* < 0.0001) (Fig. 1G–I, Supplementary Fig. 1D). Using confocal microscope imaging of MM cells, we found that the fluorescence-conjugated nanoparticles prominently localized to the cytosol in MM.1S cells and migrated toward the perinuclear regions with longer periods of incubation (Fig. 1J). We also found a time-dependent increase in cellular internalization of BCMA-BTZ-NPs using another high BCMA-expressing MM cell line, MM.1S (Supplementary Fig. 2), and a low-expressing normal human dermal fibroblasts cell line, HDF (Supplementary Fig. 2). BCMA-BTZ-NPs were internalized more efficiently by BCMA-overexpressing cells (75.5 ± 2.3% and 29 ± 0.6% (*p* < 0.0001) for MM.1S and HDF, respectively, after 6 h of incubation), suggesting BCMA-mediated uptake.

### Evaluation of target-specific cytotoxicity of BCMA-BTZ-NPs towards MM cells and patient samples

**Fig. 2:**
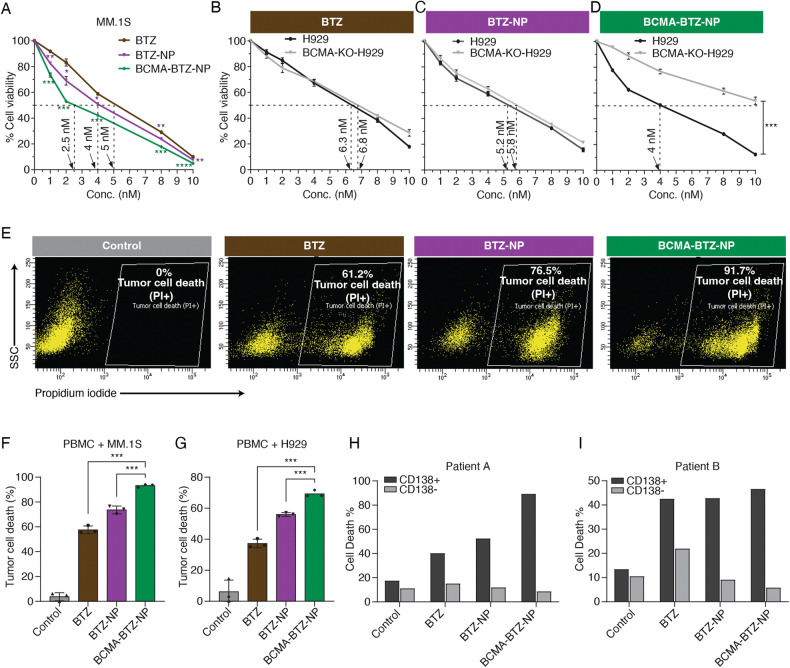
Target-specific anti-proliferative activity of BCMA-BTZ-NP in MM cell lines and patient samples. **A** Cytotoxicity of free drug BTZ, BTZ-NPs, and BCMA-BTZ-NPs in MM.1S. Cytotoxicity of **B** BTZ, **C** BTZ-NPs, and **D** BCMA-BTZ-NPs on WT-H929 and BCMA-KO-H929 cells. **E** Representative image of tumor cell death percentage, assessed by PI staining of CFSE-positive gated MM.1S cells, in 1:5 co-culture of CFSE pre-stained MM.1S cells with PBMCs treated with Vehicle Control, BTZ, BTZ-NPs, or BCMA-BTZ-NPs. Bar plot of tumor cell death determined by PI staining on **F** CFSE pre-stained MM.1S cells with PBMC as well as **G** CFSE pre-stained H929 cells with PBMC. Therapeutic efficacy of nanoparticles to target patient BMNC-derived CD138+ and CD138- cells in a 1:1 co-culture treated for 24 h. After treatment, CD138+ and CD138- cells were gated first in flow followed by assessing PI-positive cell death percentage for **H** multiple myeloma patient A and **I** multiple myeloma patient B. Mean ± SD of triplicate cultures, ***p < 0.001, **p < 0.01, *p < 0.05, significance was assessed by Student’s t-test.

After confirming the particles’ quality control criteria, we next compared the toxicity of free drug (BTZ), nano-encapsulated BTZ, and the BCMA-targeted BTZ nanoparticles on myeloma cell line MM.1S (Fig. 2A), and on PBMC isolated from a normal volunteer (Supplementary Fig. 3A) to determine any off-target effects. The highest cytotoxicity was observed for BCMA-BTZ-NPs after 24 h of treatment in MM.1S cells, with an IC_50_ value of 2.5 nM as compared to BTZ-NPs (IC_50_: 4 nM) and free BTZ (IC_50_: 5 nM). In contrast, we observed that free BTZ had the highest toxicity towards PBMC as compared to the nanoencapsulated BTZ (Supplementary Fig. 3A), suggesting the specificity of the nanoparticles towards MM cells.

We next studied the in vitro cytotoxicity of BTZ on H929 MM cells, and observed that BCMA-BTZ-NPs induced cell death more efficiently than non-targeted BTZ nanoparticles or free BTZ (*p* < 0.001): BTZ (IC_50_: 6.3 nM); BTZ-NPs (IC_50_: 5.2 nM) and BCMA-BTZ-NPs (IC_50_: 4 nM). To investigate target-specific therapeutic efficacy, we incubated WT-H929 cells as well as BCMA-KO-H929 cells with free BTZ, BTZ-NPs and BCMA-BTZ-NPs. No significant change in cytotoxicity was observed between free BTZ and BTZ-NPs on WT and KO cells (Fig. 2B, C); however, a significant difference was observed in cytotoxicity on WT and KO H929 cells treated with BCMA-BTZ-NPs (*p* < 0.001) (Fig. 2D). Importantly, BCMA-BTZ-NPs demonstrated the lowest toxicity towards BCMA-KO cells and the highest cytotoxicity against WT-H929 cells, indicating target-specific cytotoxicity.

To determine the target-specificity towards cancer cells in blood, we mimicked the blood circulation system of MM patients by mixing CFSE-stained MM cells with PBMCs from a normal donor at a ratio of 1:5, respectively, and we incubated the cell mixture with either free BTZ or nanoparticles. We first gated (Supplementary Fig. 3B) on the CFSE-positive cell population and evaluated tumor cell death using propidium iodide (PI) (Fig. 2E). BCMA-BTZ-NPs triggered the highest percentage of MM cell death in both types of cells: PBMC with MM.1S (93.1 ± 1% vs. 73.6 ± 2.6% for Targeted vs. Non-targeted, *p* < 0.001)(Fig. 2F), as well as PBMC mixed with H929 (69.2 ± 1.8% vs. 55.93 ± 1% for Targeted vs. Non-targeted, *p* < 0.001) (Fig. 2G), as compared to BTZ-NP, and free BTZ. In fact, when we mixed CD138+ and CD138- cell populations from MM patients’ bone marrow samples and incubated them with the nanoparticles, we found that the BCMA-BTZ-NPs killed the lowest percentage of non-tumor (CD138-) cells and the highest percentage of MM (CD138 + ) cells (Fig. 2H, I).

### BCMA-targeted bortezomib nanoparticles efficiently induce both the intrinsic and extrinsic pathways of apoptosis

**Fig. 3:**
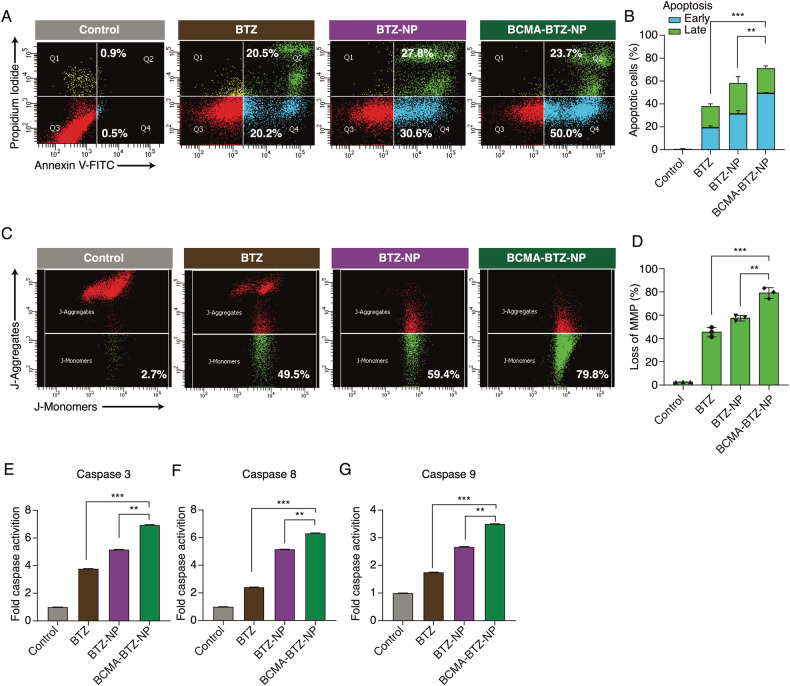
Targeted nanoparticles induce the highest percentage of apoptotic cell death. Apoptotic cell death induction in MM.1S cells after treatment with BTZ, BTZ-NPs and BCMA-BTZ-NPs for 24 h, measured by Annexin V/PI assay: **A** representative FACs images and **B** bar plot. The percentage of mitochondrial membrane depolarization (MMP) was measured by JC1 staining: **C** representative FACs images and **D** bar plot. Evaluation of Caspase Activities in MM.1S cells: Bar diagram showing progressive increased activity in BTZ, BTZ-NPs and BCMA-BTZ-NPs, respectively. for **E** Caspase 3, **F** Caspase 8, and **G** Caspase 9 after 24 h of treatment. Mean ± SD of triplicate cultures, ***p < 0.001, **p < 0.01, significance was analyzed by Student’s t-test.

To further evaluate in vitro therapeutic efficacy, we performed a cytotoxicity assay on MM cells and observed the highest potency by BCMA-BTZ-NPs. As BTZ induces cell death through apoptosis, we evaluated the mechanism of cell death induction by monitoring the apoptotic cell percentage of MM.1S and H929 cells treated with free BTZ and nanoparticles using Annexin V/PI staining. Consistent with cell growth inhibition, the highest apoptotic cell death percentage was caused by BCMA-BTZ-NP treatment for 24 h in both MM.1S (71.33 ± 1.8% vs. 58.37 ± 3%, *p* < 0.01 for Targeted vs. Non-Targeted) (Fig. 3A, B); and H929 cells (31.4 ± 3.1% vs. 19.47 ± 2.2%, *p* < 0.01 for Targeted vs. Non-Targeted) (Supplementary Fig. 4A, B). Mitochondrial depolarization is a hallmark of the intrinsic apoptotic pathway, and we observed that BCMA-BTZ-NPs significantly outperformed non-targeted nanoparticles and free BTZ, evidenced by JC1 staining in MM.1S (79.06 ± 3.7% vs. 57.4 ± 1.9%, *p* < 0.01 for Targeted vs. Non-Targeted) (Fig. 3C, D) and in H929 (47.23 ± 1.9% vs. 31.26 ± 0.7%, *p* < 0.001 for Targeted vs. Non-Targeted) (Supplementary Fig. 4C, D). Similarly, enhanced activation of caspases 8, 9, and 3 was observed after treatment with BCMA-BTZ-NPs compared to unconjugated nanoparticles or free BTZ, indicating that BCMA-BTZ-NPs more efficiently triggered activation of both intrinsic and extrinsic apoptotic pathways in MM.1S (*p* < 0.01) (Fig. 3E–G) and H929 cells (*p* < 0.01) (Supplementary Fig. 4E–G).

### Nanoparticles induce cell death even in acquired BTZ-resistant cells with high expression of PgP and within the MM tumor microenvironment

**Fig. 4:**
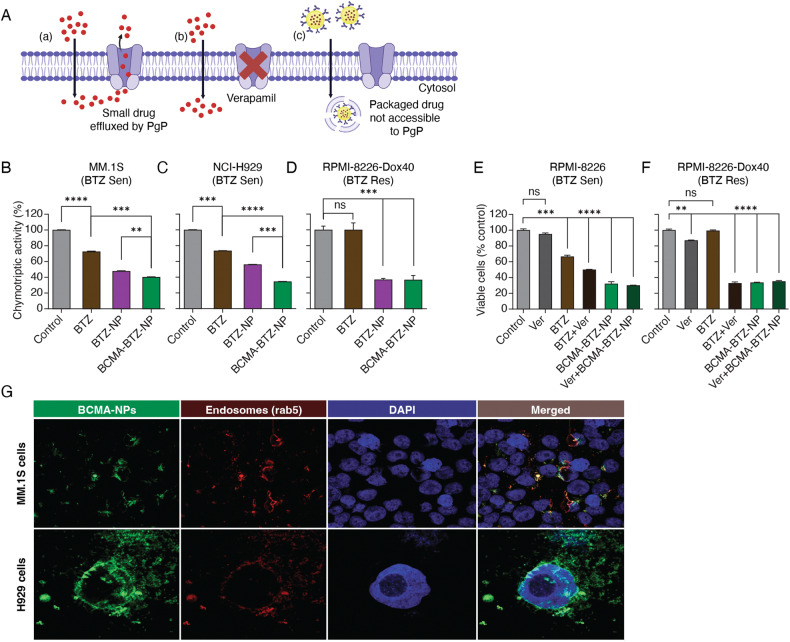
Nanoparticles can overcome BTZ resistance by using receptor-mediated endocytosis. **A** Schematic representation of (a) free BTZ cellular uptake by simple diffusion and immediate efflux out by PgP pump. (b) When PgP pump is blocked by verapamil, then free BTZ stays in cytosol. (c) Nanoparticulated drug delivery system avoids PgP-mediated drug efflux. Inhibition of chymotryptic activity was measured after treatment with BTZ, BTZ-NPs, and BCMA-BTZ-NPs in BTZ-sensitive **B** MM.1S and **C** H929 cells, as well as BTZ-resistant **D** RPMI-8226 Dox-40 cells. Cytotoxicity was measured on **E** BTZ-sensitive RPMI-8226 and **F** BTZ-resistant RPMI-8226 Dox-40 myeloma cell lines after treatment with PgP inhibitor verapamil, alone and in combination with BTZ and BTZ nanoparticles. **G** Confocal images of MM.1S cells (Upper panel) and H929 (Lower panel) showing co-localization of nanoparticles with early endosomes. Mean ± SD of 2-5 independent experiments performed in triplicate, ns not significant, ****p < 0.0001, ***p < 0.001, **p < 0.01, *p < 0.05, significance determined by Student’s t-test.

We next measured the inhibition of chymotryptic proteasomal activity after treatment with free BTZ or nanoparticles in both BTZ-sensitive and BTZ-resistant MM cells. Although small molecule free drugs may immediately be effluxed by the cells overexpressing the PgP pump before diffusing inside the cell, nanoparticles conventionally enter the cell by receptor-mediated endocytosis, thereby avoiding the PgP pump (Fig. 4A). As expected, BCMA-BTZ-NPs were found to induce significantly greater inhibition of chymotryptic activity than free BTZ or non-targeted nanoparticles in BTZ-sensitive MM.1S (*p* < 0.001 for free drug vs. Targeted and *p* < 0.01 Targeted vs. Non-Targeted) and H929 (*p* < 0.0001 for free drug vs. Targeted and *p* < 0.001 Targeted vs. Non-Targeted) cell lines (Fig. 4B, C). To examine whether BCMA-BTZ-NPs overcome acquired BTZ resistance, we treated the RPMI-8226-Dox-40 and AMO-I (BTZ-Res) cells (Supplementary Fig. 5A) and observed that BCMA-BTZ-NPs were still effective in both BTZ-resistant cell lines (36.66 ± 1.8% chymotryptic activity for RPMI-Dox40 MM resistant and 40.29 ± 1.0% for MM.1S MM sensitive cell line); in contrast, free BTZ showed no inhibitory effect on chymotryptic activity (100.01 ± 4.6% chymotryptic activity for RPMI-Dox40 MM Res) (Fig. 4D). These results suggest that there is a difference between free BTZ and BCMA-BTZ-NPs in drug cellular internalization and accessibility in BTZ-resistant cells. A similar finding was observed in a cytotoxicity assay with nanoparticles in BTZ-resistant cell lines (Supplementary Fig. 5B, C).

The overexpression of PgP can lead to the development of drug resistance [30]. In the BTZ-resistant RPMI-8226-Dox-40 cells, PgP expression is significantly increased compared to the parental RPMI-8226 cells (Supplementary Fig. 5D, E). To confirm the role of PgP in BTZ-resistant RPMI-Dox40 cells, we used a PgP blocker verapamil in combination with either free BTZ or BTZ-loaded nanoparticles to treat BTZ-sensitive RPMI-8226 and BTZ-resistant RPMI-Dox40 cells. Although there was a significant effect of verapamil on cytotoxicity (Fig. 4E, F) and ROS generation (Supplementary Fig. 6A, B) when used in combination with free BTZ in RPMI-Dox40 cells (*p* < 0.0001 for free BTZ vs. Ver+BTZ combination treatment), no significant effect was observed when used with BTZ-loaded nanoparticles in both BTZ-sensitive and RPMI-Dox40 cells (Fig. 4E, F). Consistent with receptor-mediated endocytosis, we found that Alexa-488-conjugated BCMA nanoparticles co-localized with Rab-5-stained endosomes (Fig. 4G). Taken together, these results suggest that the nanoparticles avoid the inhibitory effect of the PgP pump by using the endocytosis pathway for cellular internalization and diffusion.

The multiple myeloma bone marrow microenvironment plays a crucial role in disease progression, specifically the stromal cells promote drug resistance and MM cell proliferation [3, 24]. So, we compared the therapeutic effect of free drug, BTZ, BTZ-NP and BCMA-BTZ-NP on MM.1S monoculture, as well as co-culture of MM.1S with stromal cells derived from MM patients. Interestingly, we found that nanoencapsulated BTZ and BCMA-targeted BTZ-NP successfully overcame the stromal-induced drug resistance (Supplementary Fig. 7A, B). Another critical obstacle for BCMA-targeted therapy is the presence of soluble BCMA (sBCMA) in MM patient plasma. Therefore, we tested the effect of exogenous recombinant BCMA (0–1000 ng/ml) on the effectiveness of BTZ, BTZ-NP and BCMA-BTZ-NP at their IC_50_ dose. We observed no effect on BCMA-BTZ-NP, even at 1000 ng/ml plasma concentration of sBCMA (Supplementary Fig. 8A, B). Furthermore, we measured the effect of higher concentrations of sBCMA (0-25 µg/ml) (Supplementary Fig. 8C, D) and found only a modest inhibitory effect.

### The nanoparticles induce ICD

**Fig. 5:**
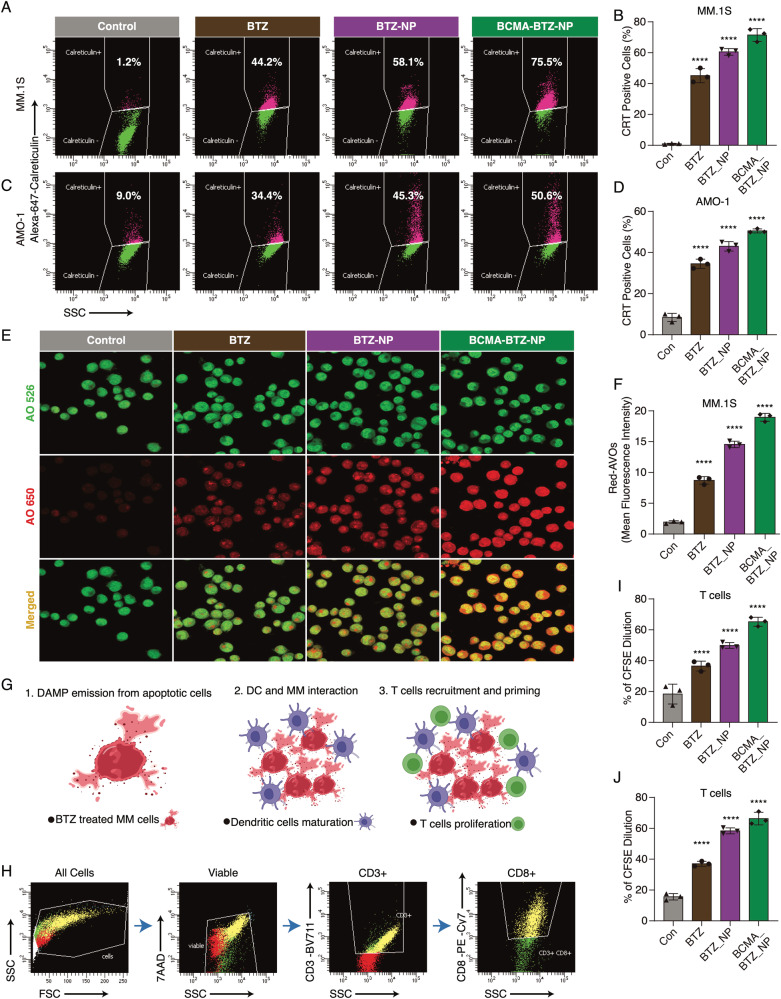
Targeted nanoparticles improve ICD, T-cell proliferation, and activate autophagic pathway. Calreticulin cell surface expression measured on **A** MM.1S with **B** respective bar plot and on **C** AMO-1 cells with **D** representative bar plot. **E** Detection of acidic vesicular organelles (AVOs) by red fluorescence, and respective nucleus as well as cytoplasm (neutral pH condition) by green fluorescence using acridine orange staining (AO), in control, BTZ, BTZ-NPs, and BCMA-BTZ-NPs treated MM.1S cells. **F** Quantitative representation of red-AVOs for different treatment groups performed using Image J software. **G** Schematic representation of ICD induction steps showing DAMP expression on apoptotic cancer cells after treatment with BTZ and BTZ nanoparticles that target dendritic cell maturation and antigen uptake, followed by T cell recruitment and priming in the tumor microenvironment. **H** Gating strategy shown for T cell proliferation assay investigated in CFSE pre-stained T cells co-cultured with dendritic cells and either free BTZ or nanoparticles treated MM cells for 5 days, with quantitative bar representations for **I** MM.1S and **J** AMO-1 cells. Mean ± SD of triplicate cultures, ns not significant, ****p < 0.0001, ***p < 0.001, **p < 0.01, *p < 0.05, significance analyzed by Student’s t-test.

Recently we have shown that BTZ triggers immunogenic cell death (ICD) [28]. We therefore next examined the effect of BCMA-BTZ-NPs on ICD. We measured surface calreticulin (CRT) expression, a hallmark of ICD induction, on the MM cell lines, MM.1S and AMO-1 (Fig. 5A–D). Consistent with our previous studies [28], we found that CRT expression in MM.1S cells was upregulated by free BTZ (45.13 ± 3.7%), which was significantly (*p* < 0.001) enhanced by BCMA-BTZ-NPs (71.5 ± 3.4%) (Fig. 5A, B). Since autophagy is required for immune induction during ICD, we investigated the impact of BTZ nanoparticles on the activation of the autophagic pathway. We observed a significant upregulation in autophagosome formation after treatment with BTZ-NPs and BCMA-BTZ-NPs compared to free BTZ, as assessed by acridine orange (AO) using confocal microscopy in MM.1S (Fig. 5E, F) and AMO-1 cells (Supplementary Fig. 9A, B). We further confirmed induction of the autophagosomes by quantifying autophagic flux with fluorescent probe Cyto-ID Green staining using flow cytometric analysis (Supplementary Fig. 9C, D).

Since T cell proliferation occurs after ICD (Fig. 5G), we next measured T cell proliferation by treating MM cells (MM.1S and AMO-1) with free BTZ or nanoparticles, followed by co-culture with DCs and CFSE pre-stained T cells. As shown in Fig. 5H–J, the nanoparticles significantly (*p* < 0.001) triggered T-cell proliferation compared to free BTZ in both MM.1S (free BTZ: 36.5 ± 2.6% vs BCMA-BTZ-NP: 65.2 ± 2.3%, *p* < 0.001) and AMO-1 (free BTZ: 37.03 ± 1.3% vs. BCMA-BTZ-NP: 66.33 ± 3.3%, *p* < 0.001) cells. Of note, we observed increased levels of TNFα, granzyme β, and IFNγ in cell culture supernatants after treatment (Schematic representation shown in Supplementary Fig. 10A) with nanoparticles versus free BTZ (*p* < 0.01 for free BTZ vs. BCMA-BTZ-NP in both MM cell line). These results suggest that BCMA-BTZ-NPs induce a more potent anti-tumor immune response (Supplementary Fig. 10B, C) than free BTZ.

### In vivo therapeutic efficacy of BCMA-BTZ-NPs

**Fig. 6:**
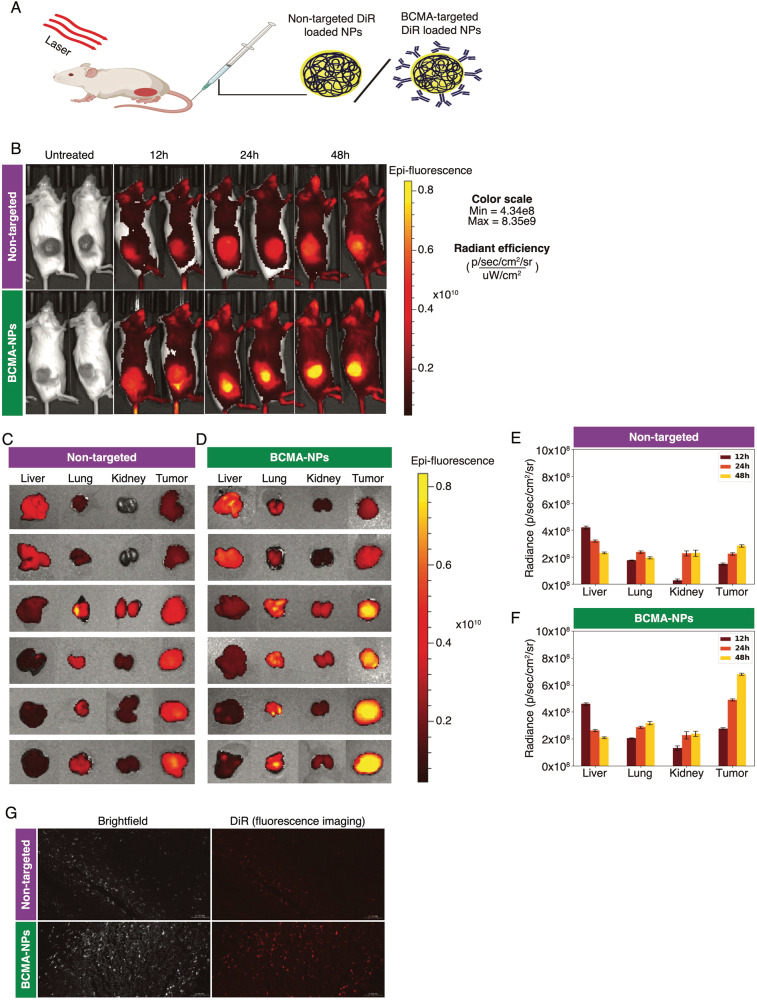
Real-time in vivo fluorescence imaging in multiple myeloma subcutaneous tumor-bearing mice at different time points. **A** Schematic representation of DiR dye- loaded non-targeted and targeted nanoparticles IV injection into multiple myeloma subcutaneous tumor-bearing mice model. **B** In vivo accumulation of DiR dye-loaded non-targeted (upper panel) and BCMA-targeted (lower panel) nanoparticles monitored at 12 h, 24 h, and 48 h. Bio distribution of DiR-dye loaded **C** non-targeted and **D** targeted nanoparticles to liver, lung, kidney, and tumor isolated 12 h, 24 h, and 48 h after IV injection, with quantitative assessment for **E** non-targeted and **F** targeted group. The respective fluorescence intensity was measured with the unit Radiance (p/s/cm2 /sr) for Region of Interest (ROI) quantified using AMI viewer image software. **G** In vivo tumor tissue localization of DiR-loaded non-targeted (upper panel) and BCMA-targeted (lower panel) nanoparticles was assessed by fluorescence imaging of tumor tissue histology from tumors extracted 48 h after IV injection. Each animal image shown here represents a single replicate from the corresponding group. Mean ± SD of 2-5 independent experiments performed in triplicate. Each organ image represents a single replicate extracted from the corresponding mouse group.

We next evaluated the target-cell selectivity of BCMA-BTZ-NPs in vivo. To monitor the real-time accumulation of BCMA-BTZ-NPs at the tumor site and in other organs, we live-imaged human MM cell xenografted NSG mice injected with DiR-loaded non-targeted BTZ-NPs and BCMA-BTZ-NPs (Schematic representation, Fig. 6A). We observed that BCMA-BTZ-NPs accumulated more efficiently at the tumor site in a time-dependent fashion compared to non-targeted BTZ-NPs (Fig. 6B).

**Fig. 7:**
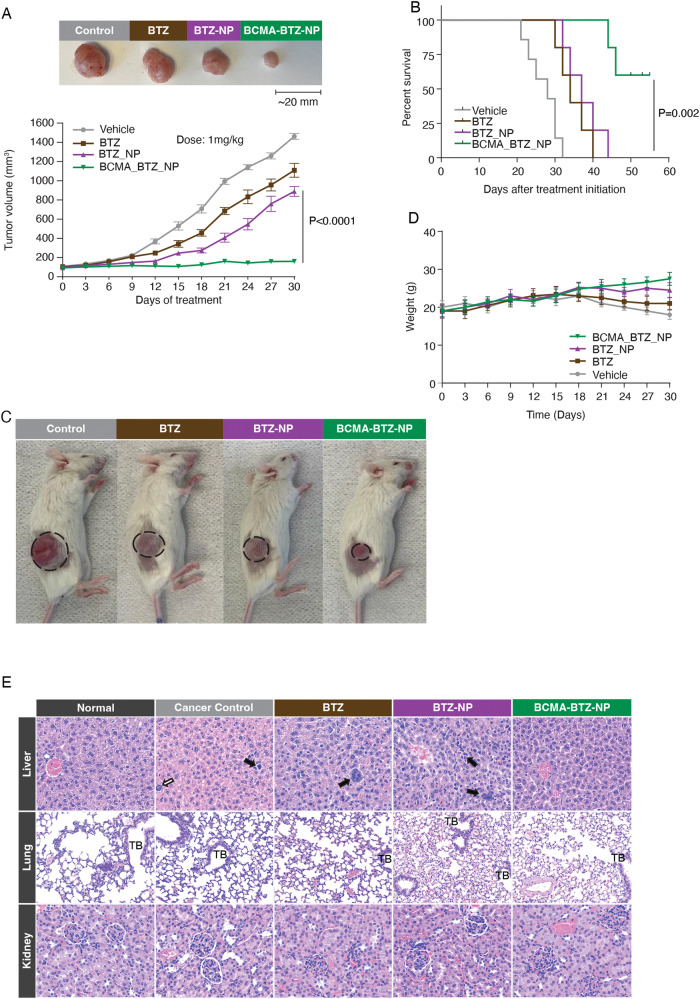
In vivo therapeutic efficiency of targeted and non-targeted nanoparticles evaluated in MM NSG mouse model. Therapeutic effect of vehicle control, BTZ, BTZ-NPs, and BCMA-BTZ-NPs on myeloma xenograft growth and survival. **A** BCMA-BTZ-NPs significantly delayed tumor growth as compared with BTZ-NPs (*p* < 0.0001). **B** BCMA-BTZ-NPs enhanced survival with respect to non-targeted NPs (*p* < 0.01) and free drug (*p* < 0.01), as shown in Kaplan-Meier survival plot. **C** Images of representative mice from different groups and respective tumor from each group treated with vehicle, free drug BTZ, BTZ-NPs and BCMA-BTZ-NPs. **D** Body weight for different treatment groups was monitored over the indicated time periods. **E** Analysis of histopathological morphology, by HE staining of liver, lung, and kidney extracted from mice who received different treatments to compare treatment effect on major organs. (Magnification of each panel 40×). Mean ± SD of 2-5 independent experiments performed in triplicate, ns not significant, ****p < 0.0001,***p < 0.001, **p < 0.01, *p < 0.05, significance analyzed by one-way ANOVA followed by Tukey’s post hoc test.

We resected and imaged the organs after injection of DiR-loaded non-targeted BTZ-NPs (12 h, 24 h, and 48 h) and found that BCMA-BTZ-NPs had enhanced accumulation in the tumor site than non-targeted BTZ-NPs (Fig. 6C–F). These results suggest that BCMA-BTZ-NPs predominantly target MM cells in vivo. We further studied the tumor tissue uptake of nanoparticles by fluorescence imaging of tumor tissue histology sections 48 h after DiR dye-loaded targeted and non-targeted particle IV injection. Preferential deposition of the nanoparticles in tumor tissue was observed for BCMA-BTZ-NPs as compared to non-targeted BTZ-NPs (Fig. 6G). Interestingly, the fluorescence intensity of the tumor’s region of interest (ROI) was 6.8 ± .86 ×10^8^ p/sec/cm^2^/sr as compared to the liver’s 2.1 ± .86 ×10^8^ p/sec/cm^2^/sr 48 h after injection of BCMA-BTZ-NP therapy. We next examined the therapeutic efficacy of BCMA-BTZ-NPs and non-targeted BTZ-NPs in the MM plasmacytoma model developed in NSG mice. We treated mice for 4 weeks with free BTZ, non-targeted BTZ nanoparticles, or BCMA-BTZ-NPs. The free BTZ was administered intravenously at 1 mg/kg per week and both Non-targeted BTZ-NPs and BCMA-BTZ-NPs were administered with the same treatment protocol and equivalent dose to the free BTZ. Non-targeted BTZ-NPs demonstrated greater tumor growth inhibition than free BTZ; however, BCMA-BTZ-NPs induced the highest tumor growth reduction (*p* < 0.0001 for Non-Targeted vs. Targeted) (Fig. 7A), associated with the longest survival (*p* < 0.01 for Non-Targeted vs. Targeted) (Fig. 7B), and without effects on host condition (Fig. 7C), or body weight (Fig. 7D).

We further evaluated the systemic toxicity of free BTZ, non-targeted BTZ-NPs, and BCMA-BTZ-NPs in major organs including liver, lung, and kidney, resected from the mice at the end of the experiment. We examined pathological changes in those organs from each cohort by hematoxylin and eosin (H&E) staining (Fig. 7E). Within the liver of the control, free drug (BTZ) and non-targeted BTZ-NPs treated groups, the parenchyma was infiltrated mainly within the sinusoids by a moderate number of rounds to oval cells, occasionally showing multinucleation and mitotic activity (neoplastic cells). This feature was not observed in the normal and BCMA-targeted groups. In cancer control, few hepatocytes showed prominent karyomegaly. Karyomegaly (karyocytomegaly), a reflection of hepatocyte polyploidy that occurs when there is duplication of nuclear material in the absence of cytokinesis. Karyocytomegaly and anisokaryosis are normal incidental findings, especially in older mice, but can also be induced by xenobiotics. Within the lungs and kidneys, no important changes were observed between the experimental groups. Alveolar histiocytosis (alveolar histiocytosis) was present in most of the individuals including controls, and in this context considered an incidental/background change. The liver tissue morphology and integrity in the targeted group were similar to the morphology of the healthy controls, suggesting minimal off-target toxicity for the BCMA-targeted nanotherapy.

## Discussion

BTZ-based combination treatments are standard treatment options in MM. Despite its impressive anti-myeloma activity in MM patients, BTZ has unfavorable off-target effects that limit its long-term clinical use [31]. To address this issue, we generated nanoparticulated BTZ (BTZ-NPs) to avoid dose-related toxicity by improving drug retention and ensuring drug accumulation at the tumor site [32, 33]. In MM, several receptor-targeting nanoparticulated drug delivery systems have been tested. For example, Swami et al. developed alendronate-targeted PLGA-PEG nanoparticles. Although these nanoparticles accumulated in the bone marrow microenvironment, they did not specifically target MM cells [34]. In addition, Puente et al. reported CD38-targeted BTZ-loaded chitosan nanoparticles that targeted MM cells and overcame the dose toxicity of free BTZ [24]. However, CD38 is expressed on various types of cells, including MM cells, activated T cells, and NK cells, which may increase the risk of unfavorable side effects. It has also been shown that P-selectin glycoprotein ligand-1 (PSGL-1)-targeted BTZ and ROCK inhibitor-loaded liposomes are more effective than free drugs or non-targeted therapy; however, they did not produce a significant difference in therapeutic efficacy in in vivo studies [35]. Since PSGL-1 is ubiquitously expressed on different types of cells, it may not be a suitable ligand for targeted drug delivery. Preferably, a cell surface molecule specifically expressed on MM cells needs to be employed. Therefore, we selected BCMA, which is highly and selectively expressed on MM cells. The BCMA-BTZ-NPs showed remarkable efficacy not only in MM cell lines but also CD138+ primary MM cells from patients, where they enhanced MM cytotoxicity with minimal effect CD138- cells compared to free BTZ. These results suggest that the anti-MM effect of BCMA-BTZ-NPs is selective and that BCMA-BTZ-NPs will have an improved therapeutic index.

The development of drug resistance is a major problem that affects patient outcome. PgP is a well-known molecule that mediates drug resistance in different types of cancers [36–38]. Indeed, Sam et al. reported that BTZ-resistant MM cells express higher levels of PgP (MDR-1), and that its inhibition by verapamil, a calcium channel blocker, can restore the sensitivity of the cells to BTZ [30]. Similar results have been reported in leukemic cells [39]. In this study, we confirmed that verapamil significantly enhances the cytotoxicity of BTZ in BTZ-resistant RPMI-Dox40 cells with high expression of PgP. Importantly, BCMA-BTZ-NPs were even more effective than BTZ-verapamil combination treatment in RPMI-Dox40 cells. Our results suggest that BCMA-BTZ-NPs are predominantly processed through the endocytic pathway, which is distinct from the ordinary drug diffusion mechanism that helps to overcome PgP-mediated drug resistance [40]. This is because the nanoparticles enter the cell encapsulated in a vesicle, which is then digested by a lysosome, thereby keeping the drug away from the cell-surface-situated PgP. BCMA-BTZ-NPs may therefore overcome this mechanism of drug resistance.

Additionally, stromal cells in the MM tumor microenvironment help MM cells develop drug resistance and prolong MM cell survival, but we found that nanoencapsulated BTZ and BCMA-targeted BTZ-NP therapy could overcome this effect of the stromal microenvironment. Moreover, we found that our BCMA-targeted nanotherapy can efficiently overcome the presence of sBCMA in patient plasma at physiological concentrations in different stages of multiple myeloma progression. Indeed, in theory, 1000 ng of sBCMA is small compared to the ~20 µg of conjugated BCMA antibody on 1 mg of nanoparticles. Yet, even higher concentrations of sBCMA (20 µg/ml) only caused a mild inhibitory effect.

**Fig. 8:**
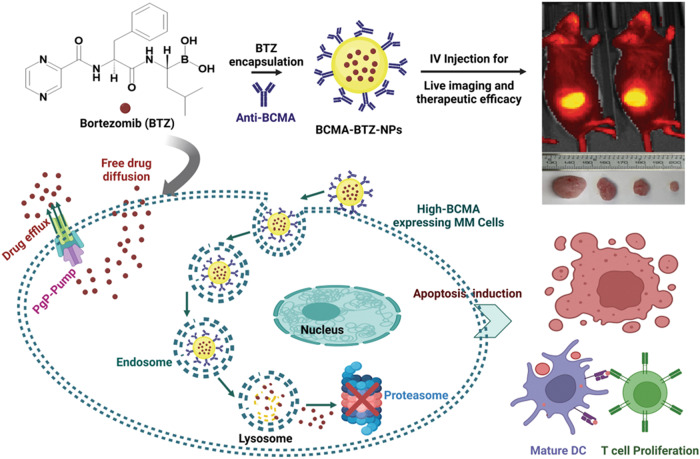
Schematic representation of BCMA-BTZ-NPs in MM. BCMA-BTZ-NPs can trigger apoptosis, improve immunogenic cell death, potentiate therapeutic efficacy even in PgP-mediated resistant cells, and demonstrate tumor-specific accumulation and cytotoxicity.

We recently reported that BTZ induces ICD, as assessed by DAMP emission and DC-mediated phagocytosis, as well as tumor cell killing by CTLs [28]. Previous studies also suggest that nanoformulated drug delivery enhances ICD induction due to slow and sustained drug release [41–43]. Therefore, we hypothesized that BCMA-BTZ-NPs may trigger ICD more potently than free BTZ. Indeed, previous studies have shown that nanoparticulated drug delivery can amplify ICD in the tumor immune microenvironment of breast cancer, pancreatic cancer, and other types of cancers [43–45]. Our studies show that BCMA-BTZ-NPs markedly augmented surface expression of calreticulin and ICD compared to free BTZ, resulting in significant upregulation of T-cell proliferation, associated with enhanced expression of granzyme β, TNFα and IFNγ. Taken together, our results indicate that BCMA-BTZ-NPs are a more efficient ICD inducer than free BTZ, acting not only as a targeted therapeutic agent but also as a modulator of anti-tumor immune response in MM (Fig. 8, schema).

Consistent with our in vitro studies, BCMA-BTZ-NPs profoundly accumulated at the tumor site in vivo and produced the highest reduction of tumor volume and prolongation of overall survival. Additionally, when we evaluated the off-target toxicity of free drug, BTZ-NPs, and BCMA-BTZ-NPs by analyzing the tissue histology of major organs of treated animals, we found remarkable pathological differences in the livers. While we observed major pathological damage in the liver of free drug-treated animals, the liver tissue histology of the BCMA-BTZ-NP-treated cohort closely resembled the morphology of the control group, indicating reduced off-target hepatotoxicity.

In summary, our studies indicate that BCMA-BTZ nanotherapy: (1) enhances therapeutic efficacy, (2) triggers immunogenic cell death, (3) reduces off-target toxicity, (4) overcomes drug resistance, and (5) accumulates at the tumor site (Fig. 8, schema). These results suggest a favorable therapeutic index and provide the framework for evaluating BCMA-BTZ nanotherapy to improve patient outcome in MM.

### Supplementary information

Final BCMA-BTZ Supplementary File

## Data Availability

The data that support the findings of this study are available on reasonable request.
