# Supplementary material for: BCMA-targeted bortezomib nanotherapy improves therapeutic efficacy, overcomes resistance, and modulates the immune microenvironment in multiple myeloma
Source: Blood Cancer J. 2023 Dec 11;13(1):184. doi: 10.1038/s41408-023-00955-y (PMC10711001; doi:10.1038/s41408-023-00955-y)
Supplement: Supplementary file 1 — Final BCMA-BTZ Supplementary File [file 41408_2023_955_MOESM1_ESM.pdf]

Supplementary Information

BCMA-targeted bortezomib nanotherapy improves therapeutic efficacy, overcomes drug resistance, and modulates the immune microenvironment in multiple myeloma

Debasmita Dutta[1], Jiye Liu[1#], Kenneth Wen[1#], Keiji Kurata[1#], Mariateresa Fulciniti[1], Annamaria Gulla[1], Teru Hideshima[1]* and Kenneth C. Anderson[1]*

[1]Department of Medical Oncology, Dana-Farber Cancer Institute, Harvard Medical School, Boston, MA, USA

Running Title: BCMA targeted bortezomib nanotherapy in multiple myeloma

[#] Contributed equally

*Co-Corresponding Author

Email address of Corresponding Authors:

teru_hideshima@dfci.harvard.edu

kenneth_anderson@dfci.harvard.edu

Materials and Methods

Cells and Materials

MM.1S, H929, RPMI 8226, and human dermal fibroblasts (HDF) cells were procured from the American Type Culture Collection (ATCC). AMO-1 was obtained from Deutsche Sammlung von Mikroorganismen und Zellkulturen (DSMZ). All cell lines were first tested for mycoplasma using the MycoAlert Mycoplasma Detection Kit (Lonza) and further validated by short tandem repeat (STR) DNA fingerprinting analysis (Molecular Diagnostic Laboratory, DFCI). All the cells except HDF cells were cultured in Dulbecco's modified Eagle's medium (DMEM) and were maintained in RPMI 1640 medium supplemented with 10% fetal bovine serum (FBS), 1× antibiotic-antimycotic, 1× GlutaMAX, and 1× Hepes at 37°C in 5% CO2.

All chemicals were purchased from Sigma (St. Louis, MO, USA), and antibodies were procured from Biolegend unless otherwise mentioned. Alexa Fluor 488 and Alexa Fluor 647 were purchased from Life Technologies (Carlsbad, CA, USA). The CellTiter-Glo®2.0 assay was obtained from Promega (Madison, USA). Caspase 3, 8, and 9 colorimetric assay kit was obtained from Abcam (USA).

Synthesis and Physical characterization of BCMA-conjugated nanoparticles

Primary emulsion was developed by dropwise addition of PBS in DCM organic phase containing PLGA and BTZ under sonication at 100W for 90-180s. The primary emulsion (w/o) was then dropwise added to 4ml of aqueous phase containing 2.5% PVA (w/v) under the same sonication condition. The BCMA antibody was conjugated on the surface of the nanoparticles using the EDC/NHS coupling reaction.

The particle size and surface charge were measured using the dynamic light scattering instrument Zetasizer Nano-ZS90 (Malvern Instruments; Westborough, MA). To determine the surface morphology and nanoparticle structure, the lyophilized powder was scanned under Transmission electron microscopy (TEM) (JEM-1000, JEOL, Tokyo, Japan). We optimized the initial drug feed to confirm the highest drug loading in the nanoformulation. Bortezomib loading in the nanoparticles was analyzed at absorbance maxima (λ = 270 nm) with an Infinite 200 Pro plate reader (Tecan). The NIR dye, DiR (1,1'-Dioctadecyl-3,3,3',3'-Tetramethylindotricarbocyanine Iodide)–loaded nanoparticles were prepared using a similar method of drug-loaded nanoparticle preparation.

The *in vitro* drug release kinetics of BTZ-nanoparticles were determined at different timepoints by collecting the supernatant from BTZ nanoparticles dissolved in PBS and incubated at 37°C under shaking to determine the drug concentration in the supernatant at λ = 270 nm using a plate reader. Antibody conjugation onto the nanoparticle surface was analyzed by orbitrap mass spectrometers (Thermo Scientific) at the Taplin Biological Mass Spectrometry Facility, Harvard Medical School. Antibody-conjugated nanoparticles and unconjugated particles were analyzed by western blotting. Additionally, we determined the covalently conjugated surface monoclonal antibody concentration of the nanoparticles by protein assay.

CellTiter-Glo cell viability assay

Cells were seeded in their respective medium (10000 cells per well, in a 100 µl final volume) in 96-well corning plates followed by treatment with free drug, different types of nanoformulations, and with verapamil. In the case of co-culturing MM cells and BMSCs, BMSCs were seeded in a 96-well corning plate, and 24h later, MM cells and

different treatment groups such as BTZ, BTZ-NP, and BCMA-BTZ-NP were added followed by co-incubation for 24h. After treatment, plates were equilibrated to room temperature after the 37°C incubator, and 100 µl of CellTiter-Glo reagent (Promega) was added to each well. Plates were gently shaken on an orbital shaker for 1 min at 500 rpm and then incubated at room temperature for 10 min. To determine the effect of soluble BCMA on cytotoxicity of BTZ, BTZ-NP, and BCMA-BTZ-NP, recombinant BCMA (Peprotech) was added to the culture media in a concentration range from 0–1000 ng/ml and 0–25 µg/ml. Luminescence was determined by a spectrophotometer (SpectraMax M3, Molecular Devices).

FACs and Confocal Microscopy-Based Assay

Cells were collected (15,000-20,000) after treatment with dye-conjugated nanoparticles and cytospun for 5 minutes at 800 rpm, followed by fixation with 4% paraformaldehyde at room temperature for 15 minutes and washing three times with 1% FBS in PBS. For detection of acidic vesicular organelles, acridine orange staining was performed following the manufacturer's protocol (Thermo Fisher Scientific). Autophagic flux was monitored by staining autophagosome and autolysosome compartments with the Cyto-ID Green fluorescent probe (Enzo Life Sciences, Farmingdale, NY), as per manufacturer's protocol. For endosome staining or PgP staining, specific dye-conjugated antibodies were used after fixation. Nuclei were co-stained with Fluoro-gel II mounting medium with DAPI (Thermo Fisher Scientific), and images were captured using the Yokogawa Spinning Disk Confocal/TIRF System and analyzed with GIMP software.

Proteasomal Activity Assay

Bortezomib-sensitive and -resistant cells were treated with free drug bortezomib BTZ-BTZ-NPs, and BCMA-BTZ-NPs, and then analyzed for chymotrypsin-like proteasome activity using the luminogenic proteasome substrate-based Proteasome-Glo Chymotrypsin-Like Cell-Based Assay Kit (Promega), as per the manufacturer's instructions. Luminescence was quantified using a 96-well plate reader.

Measurement of Apoptosis Induction, Mitochondrial Membrane Potential, and ROS generation

Apoptosis induction after treatment was evaluated by Annexin-V/PI staining and flow cytometric analysis, as previously described. The cationic lipophilic dye 5,5′,6,6′-tetrachloro-1,1′,3,3′-tetraethyl benzimidazolylcarbocyanine iodide (JC-1, Sigma-Aldrich) was used to monitor the mitochondrial transmembrane potential ($\Delta\Psi m$) following the previously mentioned procedure. ROS generation was assessed in BTZ-sensitive and -resistant cells, after incubation with BTZ, BTZ-nanoparticles, and verapamil, alone or in combination, by monitoring the hydrogen peroxide concentration using the ROS-Glo™ H_2O_2 Assay (G8820, Promega) kit following the manufacturer's protocol. Luminescence was determined using a plate reader.

Measurement of Caspases activation

Cell lysates were prepared from MM.1S and H929 cells after treatment with free drug, BTZ, BTZ-NPs, and BCMA-BTZ-NPs using RIPA buffer, and protein concentration was determined. Each sample with 150 µg of protein was aliquoted, and DTT with respective DEVD-p-NA substrate were added according to the manufacturer's protocol. Samples were mixed well and incubated at 37°C for 90 min. The OD was measured at 405 nm using a plate reader.

ELISA Assay

MM cells were either treated with free drug, different nanoformulations, or left untreated. They were co-cultured with Mo-DC and naive T-cells. The supernatant was collected to measure the secretion of Granzyme β, IFNγ and TNFα by ELISA Kit (Thermo Fisher Scientific).

MM Xenograft mice model for therapeutic efficacy study

MM plasmacytoma mice model was developed by subcutaneously injecting 5×10^6 MM.1S cells dissolved in 1:1 PBS and Matrigel mixture in NSG female mice. Health condition, body weight, and tumor burden were regularly monitored 3 days per week. The size of the tumor was monitored using an electronic caliper, and tumor volume was measured using the formula: (length × width^2) × 2^{-1} where length is greater than width. Tumor progression was monitored in all the different experimental groups along with general health condition, body weight, and survival of the animals to assess therapeutic efficacy.

Supplementary Figures

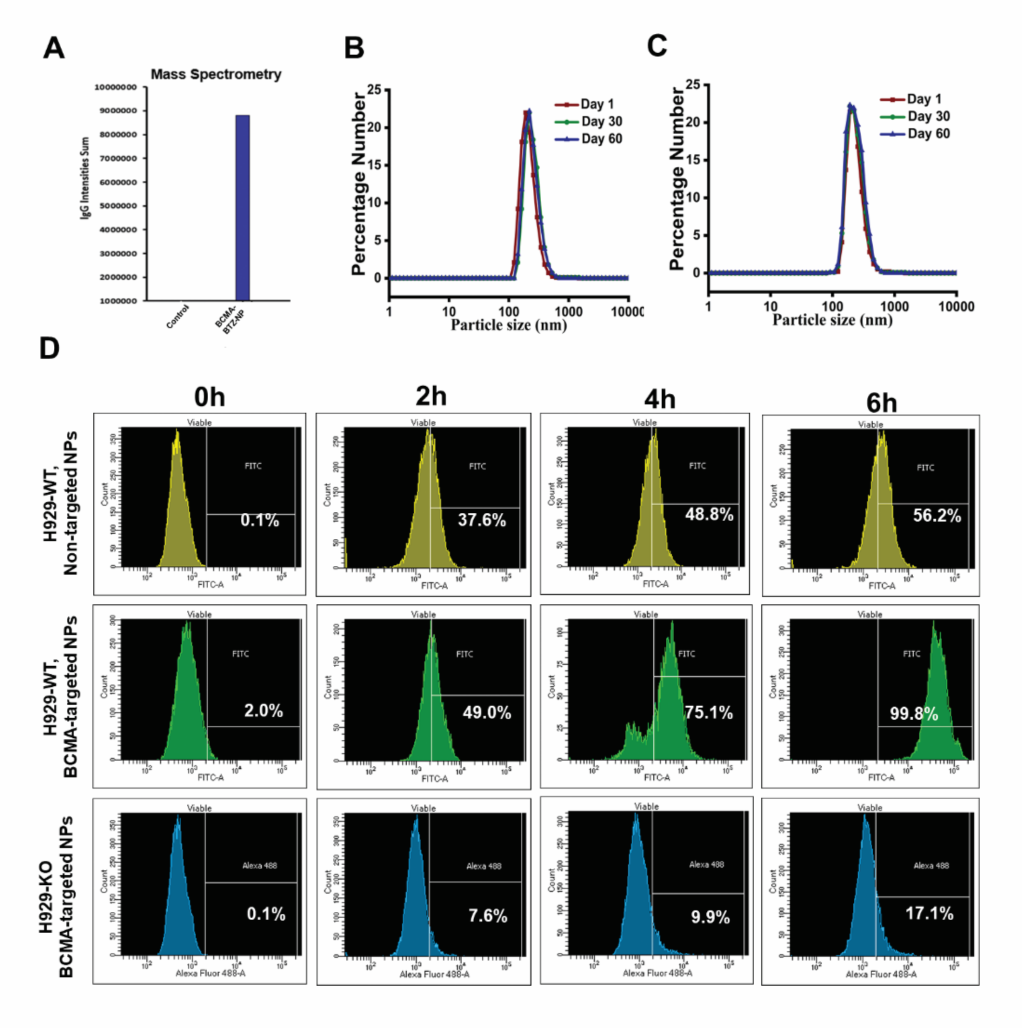

Supplementary Figure 1. Nanoparticle characterization, stability study, and cellular uptake in H929-WT and KO cells. A Mass spectrometry data revealed a significant increase in IgG intensity between antibody-conjugated and unconjugated control. Stability study for **B** targeted and **C** non-targeted nanoparticles in colloidal suspension form and under refrigerated condition was determined by monitoring Particle size distribution using DLS instrument. **D** Representative images of percentage cellular uptake monitored in WT-H929 cells after 2h, 4h, and 6h of incubation with non-targeted nanoparticles (upper panel) and BCMA-targeted nanoparticles (middle panel). Percentage cellular uptake monitored in BCMA-KO-H929 cells

after 2h, 4h, and 6h incubation with BCMA-BTZ-NPs (lower panel). Mean ± SD of 2-5 independent experiments performed in triplicate.

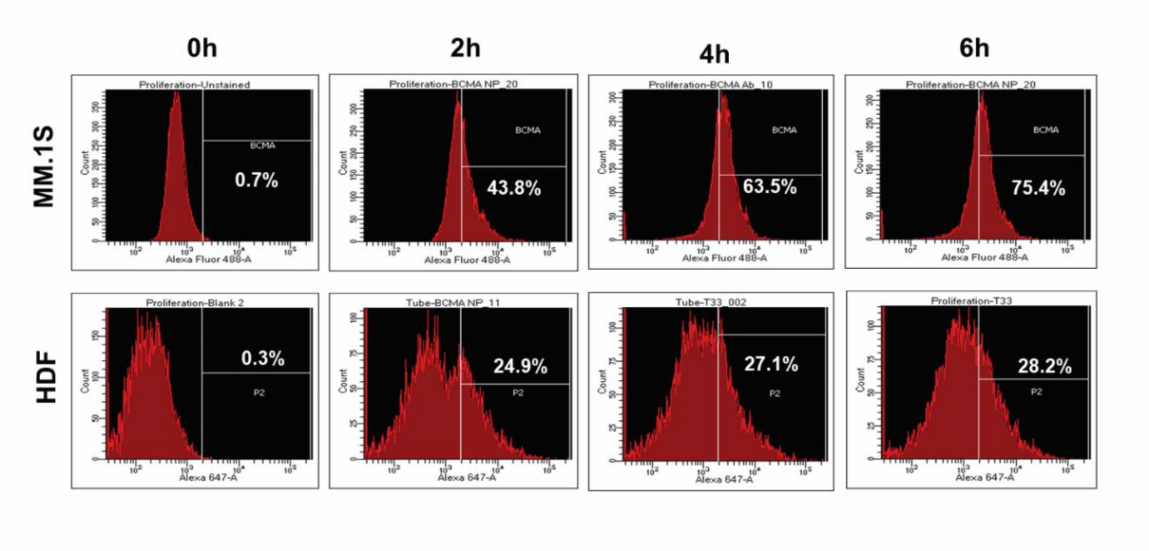

Supplementary Figure 2. Nanoparticle cellular uptake in MM.1S and Normal HDF cells. Cellular internalization percentage was studied in BCMA-high expressing MM cell line MM.1S (Upper panel) and BCMA-low expressing HDF normal cells (lower panel) after 2h, 4h, and 6h of incubation with BCMA targeted nanoparticles. Mean ± SD of triplicate cultures. FACs histograms show one of these representative images.

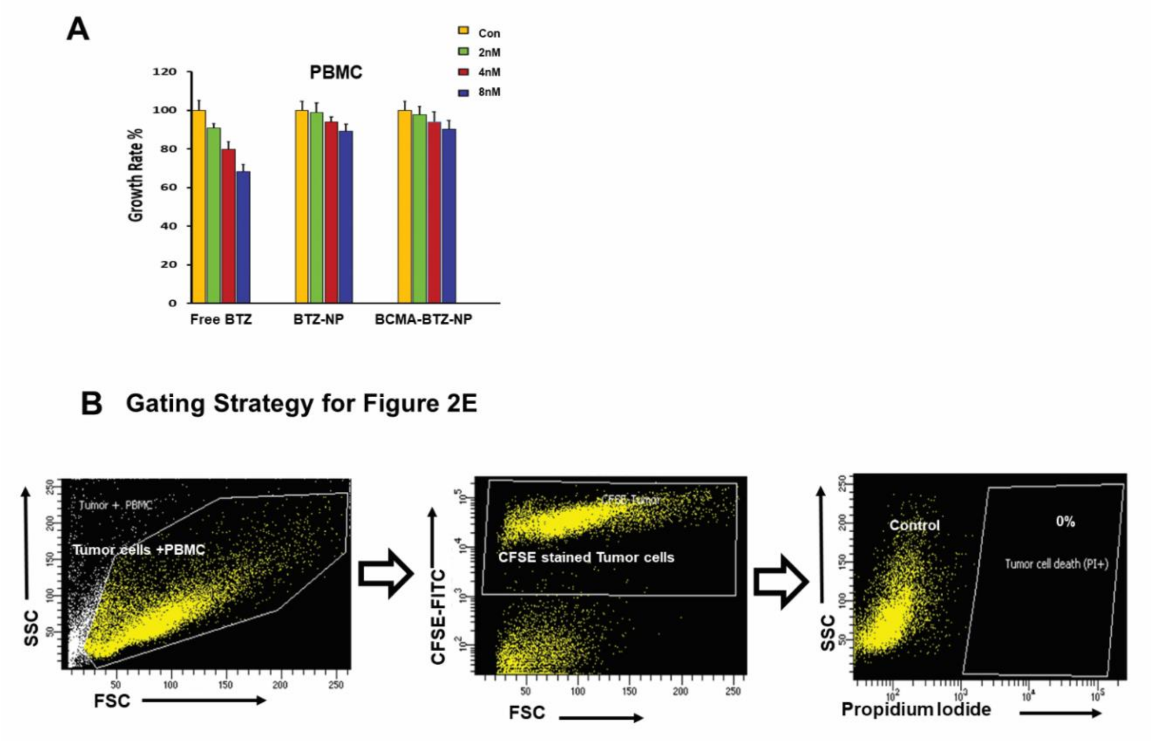

Supplementary Figure 3. Nanoparticle cytotoxicity towards PBMC and Gating strategy for assessing tumor cell death. A Cytotoxicity of free drug BTZ, BTZ-NPs and BCMA-BTZ-NPs particles was evaluated in PBMC isolated from normal donors after 24h of incubation **B** Gating strategy for tumor cell death assessed by PI staining on CFSE-positive gated MM.1S cells in 1:5 co-culture of CFSE pre-stained MM.1S cells and PBMC after treatment with Vehicle Control, BTZ, BTZ NPs, and BCMA-BTZ-NPs. Mean ± SD of triplicate cultures.

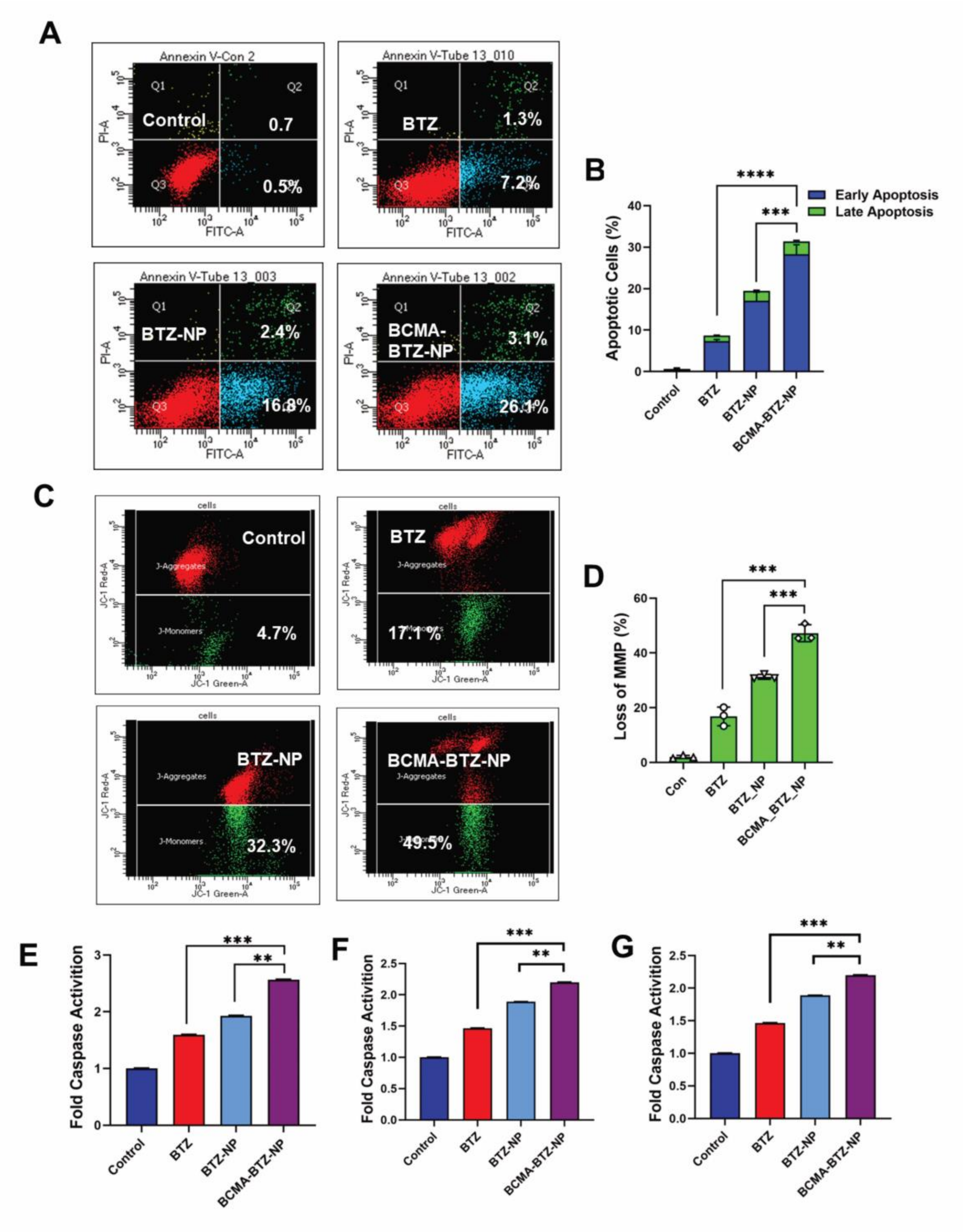

Supplementary Figure 4. BCMA-BTZ-NPs significantly increase apoptotic cell death in H929 cells. Apoptotic cell death induction in H929 cells after treatment with BTZ, BTZ-NPs and BCMA- BTZ-NPs for 24h, measured by Annexin V/PI assay: **A** representative FACs images and **B** bar plot. The percentage of mitochondrial membrane depolarization (MMP) was measured by JC1 staining: **C** representative FACs images and **D** bar plot. Evaluation of Caspase Activities in H929 cells showed progressive increased activity of **E** Caspase 3, **F** Caspase 8,

and **G** Caspase 9 after 24h of treatment with BTZ, BTZ-NPs, and BCMA-BTZ-NPs respectively. Mean ± SD of 2-5 independent experiments performed in triplicate, *** p<0.001, determined by Student's t-test.

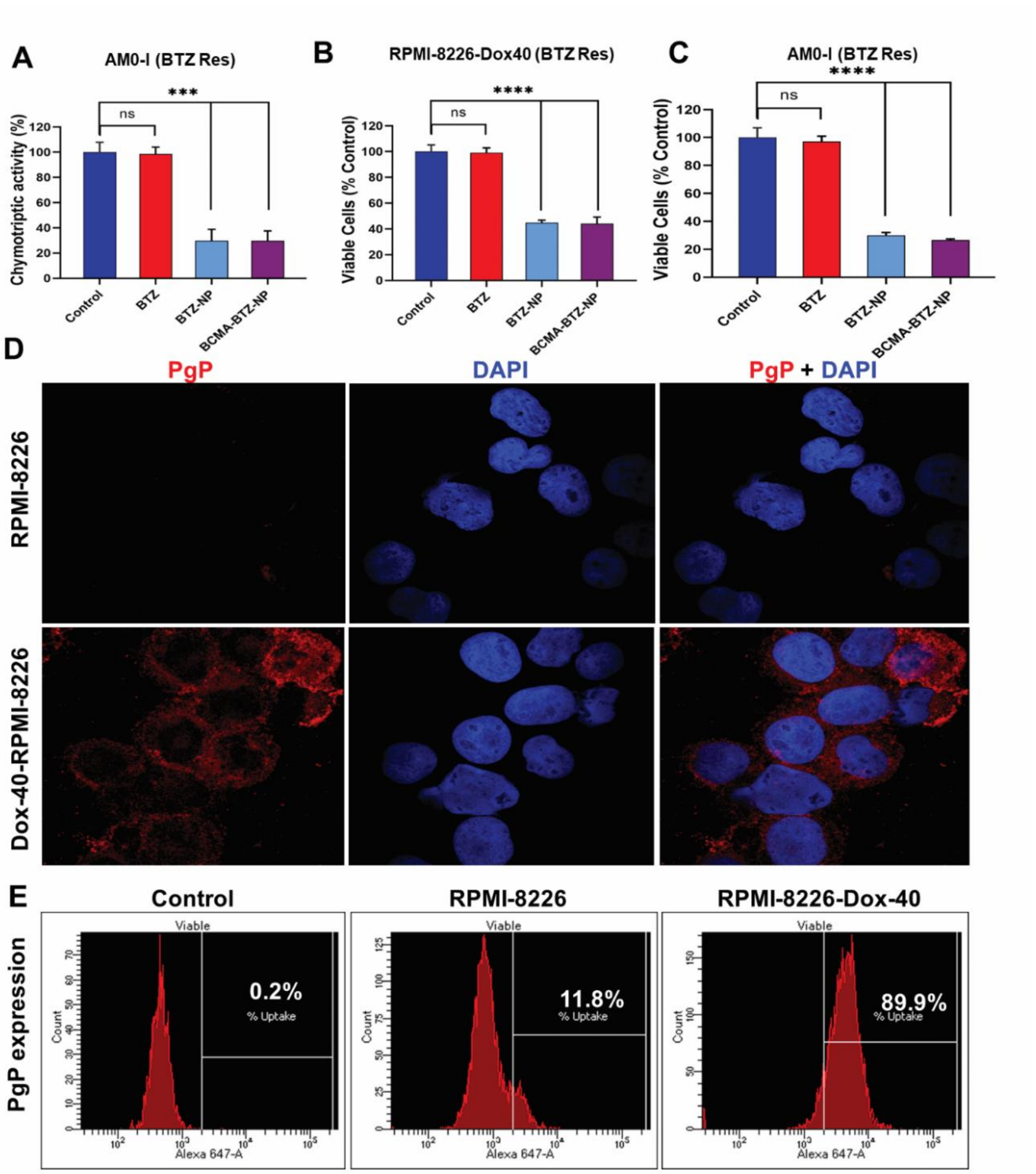

Supplementary Figure 5. Assessment of Proteasome activity in BTZ Sen and Res cells and measurement of PgP expression level. A Inhibition of chymotryptic activity was measured after treatment with BTZ, BTZ-NP, and BCMA-BTZ-NP of BTZ-resistant AMO-1 BTZ-Res cells. Cytotoxicity of BTZ, BTZ-NP, and BCMA-BTZ-NP was evaluated in **B** RPMI-8226

Dox-40 and **C** AMO-1 BTZ-Res cells. PgP expression level was determined by **D** confocal microscopy, Scale bar, 20 µm and **E** flow cytometry in BTZ-sensitive RPMI-8226 cells and BTZ-resistant RPMI-8226-Dox-40 cells. Figures show representative images of at least three independent experiments in triplicate technical repeats.

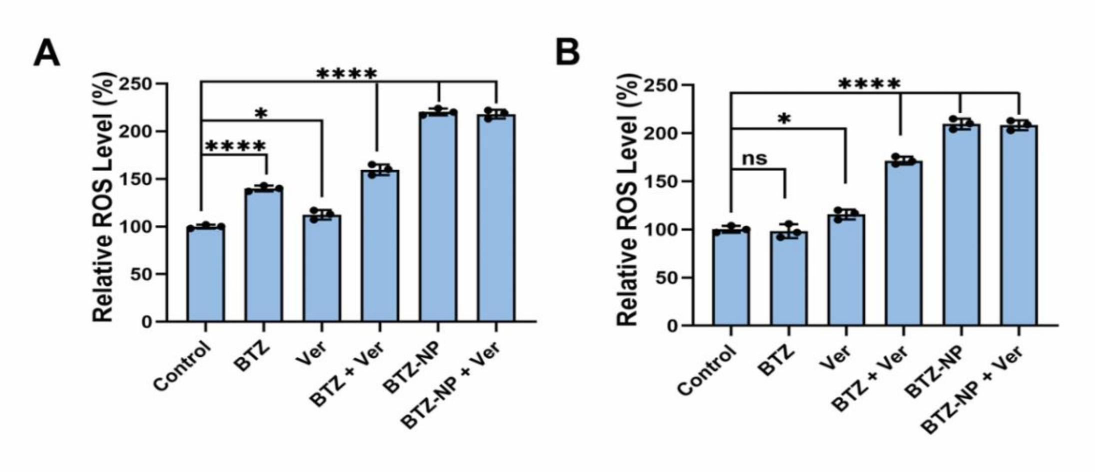

Supplementary Figure 6. Nanoparticles can overcome BTZ resistance. Intracellular ROS generation was measured in **A** BTZ-sensitive RPMI-8226 and **B** BTZ-resistant RPMI-8226 Dox-40 myeloma cell lines after treatment with PgP inhibitor verapamil, alone and in combination with BTZ and BTZ nanoparticles. Mean ± SD of triplicate cultures, **ns** for not significant, **** p<0.0001, *** p<0.001, ** p<0.01, significance determined by Student's t-test.

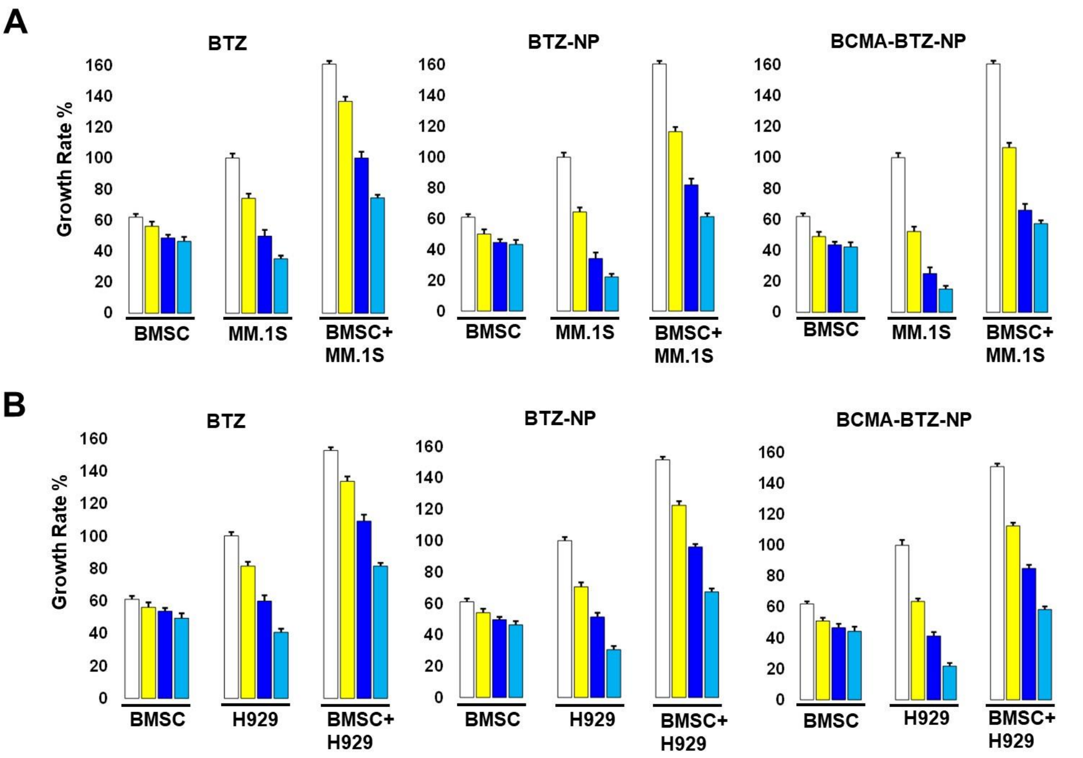

Supplementary Figure 7. Nanoparticles can efficiently overcome drug resistance in tumor microenvironment. **A** MM.1S and **B** H929 cells were incubated with culture media (white bars), 2.5 nM (yellow bars), 5 nM (dark blue bars), or 7.5 nM (light blue bars) of free BTZ, BTZ-NP or BCMA-BTZ-NP in the presence and absence of MM patient-derived stromal cells for 24h. Mean ± SD, n=2-5 independent experiments in triplicate technical repeats.

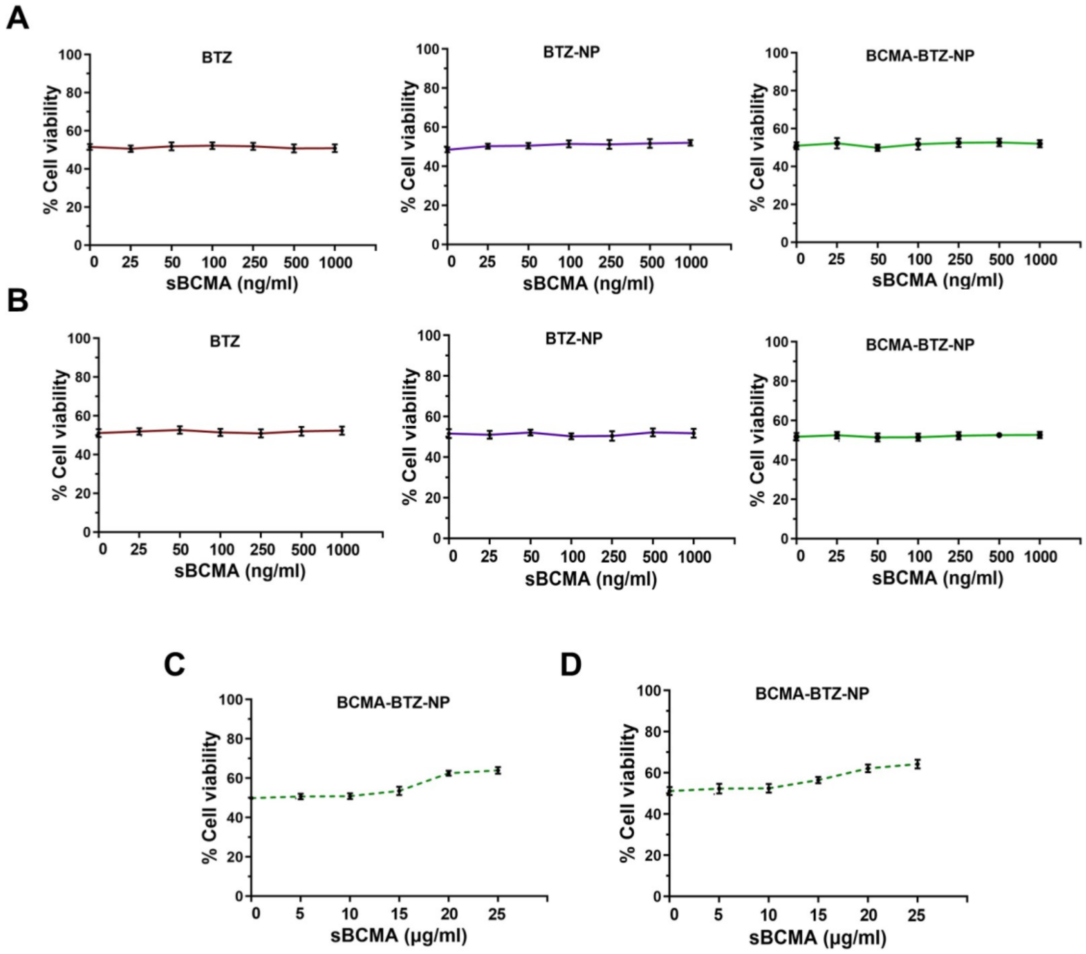

Supplementary Figure 8. Nanoparticles can effectively induce cell death in the presence of sBCMA. **A** MM.1S and **B** H929 cells were incubated with the respective IC_{50} dose of free BTZ, BTZ-NP, or BCMA-BTZ-NP for 24h in the presence of 0 to 1000 ng/ml of exogenous recombinant BCMA. **C** MM.1S and **D** H929 cells were incubated with the respective IC_{50} dose of BCMA-BTZ-NP for 24h in the presence of 0-25 µg/ml of exogenous recombinant BCMA. Mean ± SD, n=2-5 independent experiments in triplicate technical repeats.

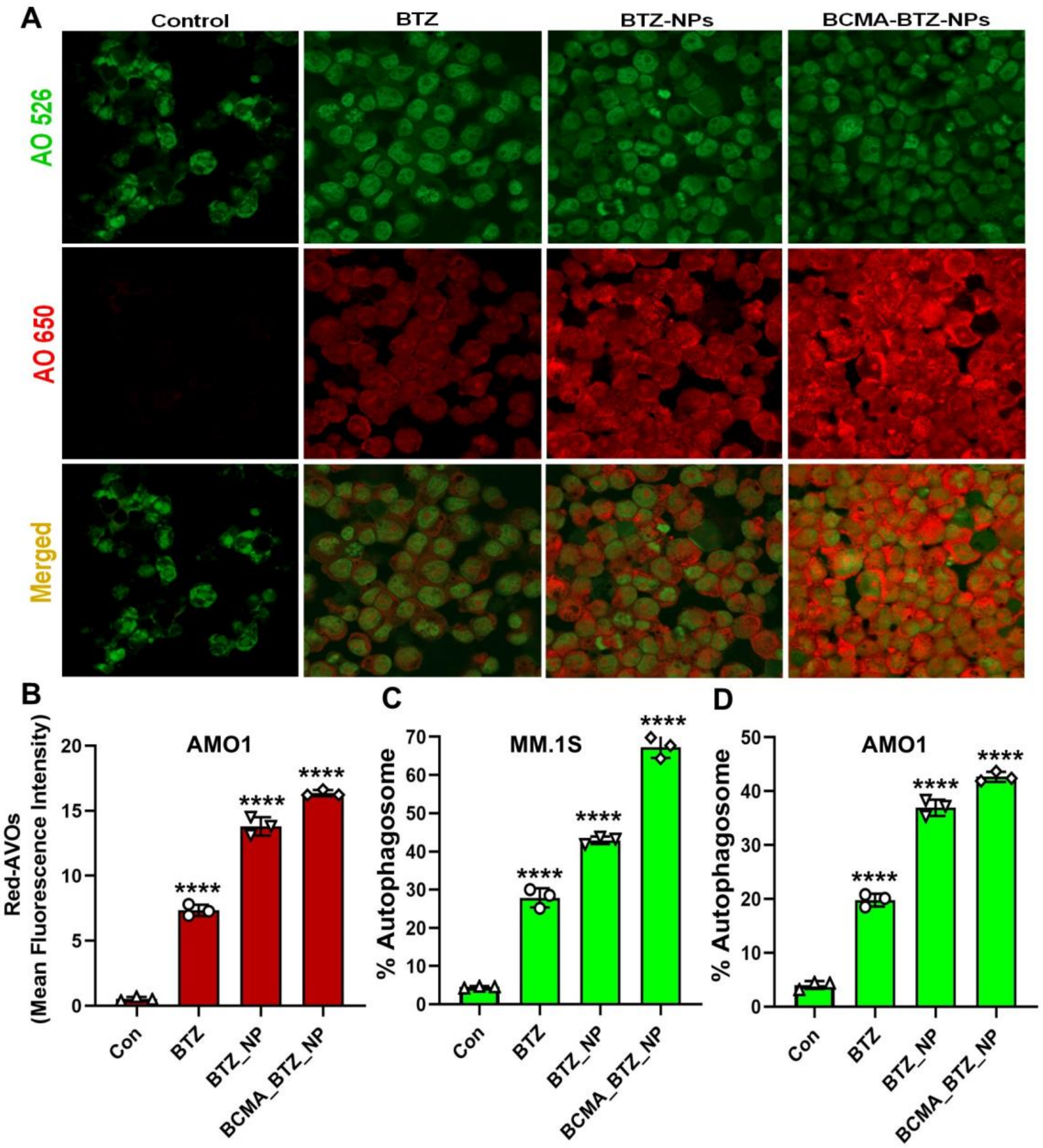

Supplementary Figure 9. Activation of autophagic pathway by BTZ and its nanoparticle formulations. A Detection of acidic vesicular organelles (AVOs) by red fluorescence and respective nucleus and cytoplasm (neutral pH condition) by green fluorescence using acridine orange staining (AO) in control, BTZ, BTZ-NP, and BCMA-BTZ-NP treated AMO-1 cells. Scale bar, 20 µm. **B** Quantitative representation of red-AVOs for different treatment groups performed using Image J software. Detection of Autophagic Flux after treatment with BTZ, BTZ-NP, and BCMA-BTZ-NP followed by fluorescent probe Cyto-ID Green staining for analysis in the green channel of flow cytometer. The percentage of autophagosomes represented

by bar diagram in **C** MM.1S and **D** AMO-1 cells. Mean ± SD of triplicate cultures, ns for not significant, **** p<0.0001, *** p<0.001, ** p<0.01, significance determined by Student's t-test.

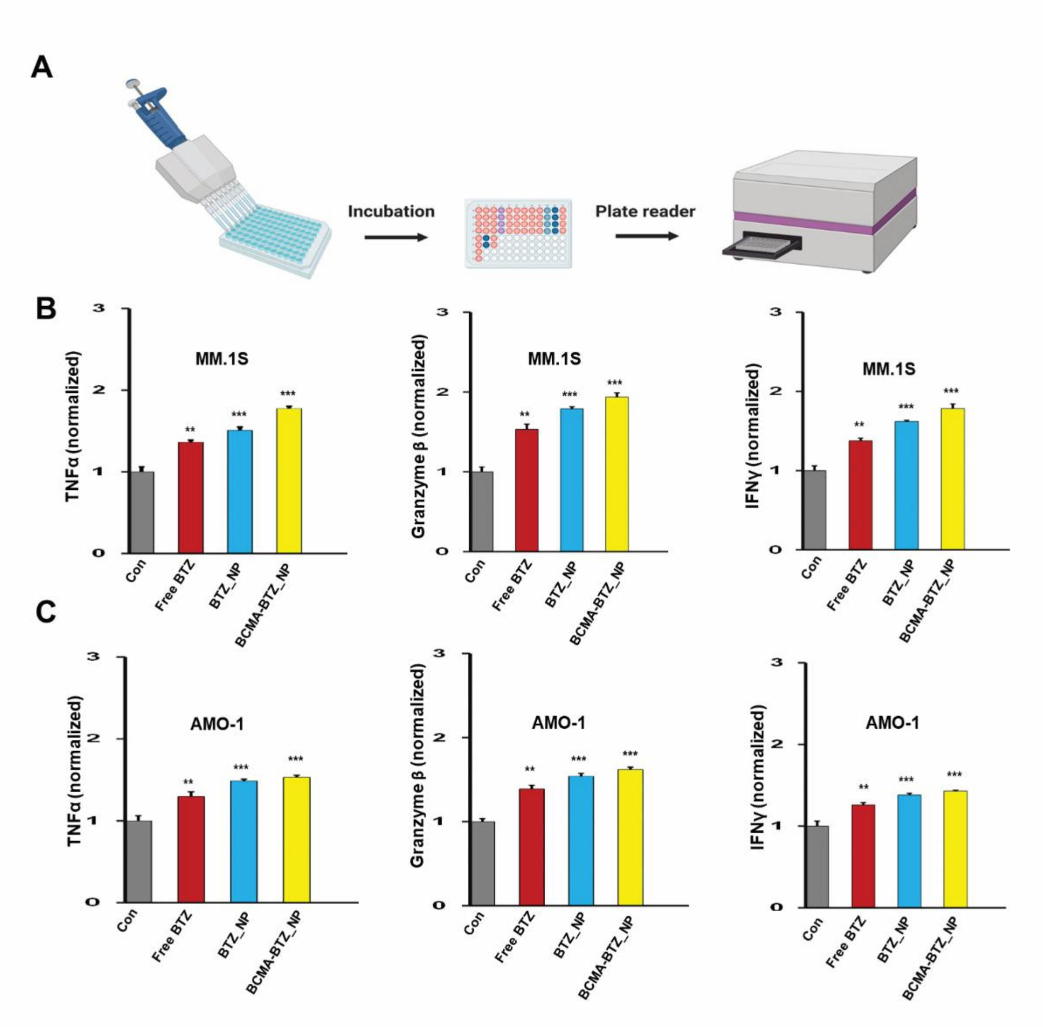

Supplementary Figure 10. Increased anti-tumorigenic cytokines level observed after targeted nanoparticle treatment. A Schematic representation of ELISA to measure cytokine profile. Anti-MM activity was measured by analysing cytokine production TNFα, Granzyme β, and IFNγ in the supernatant collected from co-cultures of dendritic cells, T cells, and **B** MM.1S or **C** AMO-1 cells pre-treated with free BTZ and different nanoencapsulated forms. Mean ± SD of triplicate cultures, ns not significant, *** p<0.001, ** p<0.01, * p<0.05, significance assessed by Student's t-test.
